# Integrated Genetic and Multi-Omic Analyses Reveal Oral Microbial Associations with Brain Structure and Alzheimer’s Disease

**DOI:** 10.3390/microorganisms14102157

**Published:** 2026-09-24

**Authors:** Yilin Liu, Xing Feng, Hongwei Dong, Tangyue Lan, Jing Wu, Chao Wang, Ximu Zhang

**Affiliations:** 1 Department of Neurobiology, School of Basic Medical Sciences, Chongqing Medical University, Chongqing 400010, China; 2 Key Laboratory of Major Brain Disease and Aging Research (Ministry of Education), Chongqing Medical University, Chongqing 400010, China; 3 College of Stomatology, Chongqing Medical University, Chongqing 401147, China; 4 Chongqing Key Laboratory of Oral Diseases, Chongqing 401147, China; 5 Chongqing Technology Innovation Center of Smart Dental Medical Devices, Chongqing 401147, China; 6 Chongqing Municipal Key Laboratory of Oral Biomedical Engineering of Higher Education, Chongqing 401147, China; 7 Chongqing Municipal Health Commission Key Laboratory of Oral Biomedical Engineering, Chongqing 401147, China; 8 Innovation and Transformation of Dental Medical Devices Engineering Research Center of Chongqing Education Commission of China, Chongqing 401147, China

**Keywords:** oral microbiome, brain structure, imaging-derived phenotypes, Mendelian randomization, multi-omics integration

## Abstract

Oral microbial dysbiosis is linked to systemic inflammatory, vascular, and metabolic perturbations, but species-level relationships with human brain structure remain poorly defined. Using two-sample Mendelian randomization, we mapped 438 of 439 oral species with genome-wide association study instruments to 1325 adult brain imaging-derived phenotypes. In total, 87 associations involving 51 taxa and 79 traits passed Benjamini–Hochberg correction across taxa within each imaging-derived phenotype. The childhood and metabolomic screens identified 196 nominal taxon–volume associations and 1118 candidate metabolite-linked paths, respectively, as exploratory findings. Spatial and single-nucleus transcriptomics localized candidate regional and cell-associated variation in brain-structure gene programs. In total, 35 taxa showed nominal genetic associations with Alzheimer’s disease. Integration with full-length oral 16S ribosomal ribonucleic acid gene sequencing from a 51-participant case–control cohort prioritized *Selenomonas infelix*, *Capnocytophaga granulosa*, and *Dialister pneumosintes*. In a secondary exploratory analysis, the three-taxon salivary model incorporating educational attainment yielded a leave-one-out cross-validated area under the receiver operating characteristic curve of 0.843. This post-selection estimate is subject to optimism, and the model remains a proof of concept requiring independent external validation. This cross-scale atlas prioritizes microbial and anatomical targets and provides a translational foundation for mechanistic validation and non-invasive neurodegenerative risk markers.

## 1. Introduction

The oral cavity is one of the body’s most densely colonized and environmentally exposed microbial interfaces. Hundreds of taxa assemble into niche-specific communities on teeth, in saliva, and across the tongue and mucosa, where host behavior, local chemistry, and immunity continuously reshape biofilm ecology [1,2,3]. When periodontal homeostasis fails, community dysbiosis sustains tissue-destructive inflammation and exposes the circulation to inflammatory mediators, microbial products, and episodic bacteremia [4]. Through immune, vascular, and metabolic signaling, oral microbial perturbation can influence systemic homeostasis well beyond the oral cavity [3,4]. This combination of dense microbial exposure, chronic inflammatory potential, and ready access to the circulation places the oral microbiome at a plausible interface with organs that are vulnerable to systemic disturbance.

These systemic properties are particularly relevant to the brain, whose structure is sensitive to chronic immune and vascular injury. Adverse periodontal health has been associated longitudinally with cognitive decline and dementia [5], and neuroimaging studies link periodontitis or tooth loss to white matter injury, hippocampal atrophy, Alzheimer’s disease (AD)-related imaging markers, and regional cortical alterations [6,7,8,9]. More recent microbiome-resolved studies connect subgingival and oral-niche communities with cortical and subcortical phenotypes and cognitive performance [10,11]. Yet these observations cannot resolve directionality: aging and vascular risk affect both oral and brain health, whereas neurodegeneration alters diet, medication use, salivary physiology, and oral self-care. Genetic studies have begun to address this problem, but have mostly represented oral disease as periodontitis rather than as specific microbial exposures; correspondingly, Mendelian randomization (MR) estimates for cortical structure, brain atrophy, and cognition remain inconsistent [12,13]. Under the instrumental-variable assumptions, MR can help address reverse causation and shared environmental confounding, while still requiring caution about instrument validity, exposure timing, and transportability [14].

Microbiota–brain research has largely centered on the gut, where regional brain associations and candidate immune, neural, and metabolic pathways are increasingly well described [15,16,17]. A recent multiscale MR study extended this framework to 43 salivary and 196 gut microbial traits across 283 brain imaging-derived phenotypes [18,19]. However, the salivary panel was derived from 16S ribosomal RNA profiles spanning phylum to genus levels and was not a uniformly species-resolved oral atlas [19]. Species-level oral microbiome genome-wide association studies (GWAS) now provide genetic instruments for hundreds of taxa [20,21], while large imaging GWAS resolve heritable variation across cortical thickness, surface area, regional volume, subcortical nuclei, and white matter [22,23,24]. This resolution matters because species within the same genus can occupy different oral niches and encode distinct metabolic and host-interaction programs. The central gap is therefore not merely whether the oral microbiome relates to the brain, but whether species-resolved signals form anatomically-specific associations and connect coherently across developmental anatomy, systemic metabolism, tissue responses, and neurodegenerative phenotypes.

Here, we built a cross-scale framework around a species-resolved MR atlas (Figure 1). We first evaluated 439 GWAS-instrumented oral microbial traits, of which 438 yielded usable harmonized estimates against 1325 adult brain imaging-derived phenotypes (IDPs), distinguishing cortical thickness, surface area, and volume. Eight childhood brain-volume traits were evaluated as a separate developmental analysis. We next screened 1400 circulating metabolites in an exploratory two-step MR to nominate candidate systemic intermediates. In parallel, we converted IDP GWAS signals into gene sets using Multi-marker Analysis of GenoMic Annotation (MAGMA) and evaluated them in bulk, spatial, and single-nucleus brain transcriptomes from mouse periodontitis models. Finally, we tested all 439 taxa against AD risk, intersected genetically prioritized species with full-length oral 16S ribosomal RNA profiles, and compared logistic regression with XGBoost in an exploratory proof-of-concept classification analysis. These complementary analyses prioritize hypotheses for mechanistic testing and independent clinical evaluation.

## 2. Methods

### 2.1. Study Design and Reporting

This study used a staged, multi-omic design to investigate links between the oral microbiome and brain structure. We first performed two-sample Mendelian randomization (MR) from species-level oral microbial traits to adult structural brain imaging-derived phenotypes (IDPs), repeated the analysis for childhood subcortical volumes, and evaluated oral microbial traits against Alzheimer’s disease (AD). We then tested plasma metabolites as potential intermediates, translated IDP genome-wide association study (GWAS) signals into gene sets, and examined these gene sets in bulk, spatial, and single-nucleus brain transcriptomic datasets from periodontitis models. Finally, MR-prioritized taxa were evaluated in a separate full-length 16S ribosomal RNA dataset and used to construct exploratory AD classifiers. MR reporting followed the Strengthening the Reporting of Observational Studies in Epidemiology Using Mendelian Randomization (STROBE-MR) statement and explanation [25,26]. Prediction analyses were reported with reference to the Transparent Reporting of a Multivariable Prediction Model for Individual Prognosis or Diagnosis plus artificial intelligence (TRIPOD+AI) statement and appraised against Prediction model Risk Of Bias ASsessment Tool plus artificial intelligence (PROBAST+AI) domains [27,28]. No prospective sample-size calculation was performed because all analyses used existing datasets. All human datasets were de-identified and publicly available. Source-study ethics and consent information are summarized in the dedicated statements below. The present secondary analysis involved no new recruitment, intervention, or animal experimentation and therefore did not require additional institutional approval.

### 2.2. Genome-Wide Association Datasets

Oral microbial exposures were obtained from the National Human Genome Research Institute–European Bioinformatics Institute (NHGRI–EBI) GWAS Catalog (GCST90710236–GCST90710674). The source study analyzed saliva-derived whole-genome sequencing data from 12,519 participants in the Simons Foundation Powering Autism Research for Knowledge (SPARK) family cohort. Species-level relative abundances were profiled with MetaPhlAn 4; 439 species present in at least 10% of participants were rank-based inverse-normal transformed before association testing. Linear mixed-model analyses performed with BOLT-LMM included sequencing batch, age terms, sex, mapped reads, microbiome principal components, and ancestry principal components [21]. Genetic effects, therefore, represent a one-standard-deviation difference in normalized taxon abundance. Adult outcomes comprised 1325 structural brain IDPs from UK Biobank (GCST90002426–GCST90003750; British ancestry participants) [23]. These outcomes included cortical thickness, surface area and volume measures, subcortical structures, cerebellum, and global or white matter phenotypes. For regional summaries, IDPs were mapped from their catalogued trait descriptions to eight prespecified groups: frontal cortex; temporal or insular cortex; parietal or cingulate cortex; occipital or visual cortex; sensorimotor cortex; subcortical or limbic structures; cerebellum or brainstem; and global, white matter, or other measures (Supplementary Table S2). Developmental analyses used eight bilateral magnetic resonance imaging (MRI) volumes—accumbens, amygdala, caudate, hippocampus, intracranial volume, pallidum, putamen, and thalamus—from 1049 European-ancestry children (GCST90296636, GCST90296639, GCST90296642, GCST90296645, GCST90296648, GCST90296651, GCST90296654, and GCST90296657) [29]. Mediation analyses used 1400 plasma metabolomic traits (1091 metabolites and 309 ratios; GCST90199621–GCST90201020; European-ancestry participants) [30]. AD summary statistics were obtained from GCST90027158, comprising 39,106 clinically diagnosed cases, 46,828 proxy cases, and 401,577 controls of European ancestry [31]. Accession-level sources are provided in Supplementary Table S1.

### 2.3. Genetic Instruments and Harmonization

For each microbial exposure, variants associated at *p* < 5 × 10^−6^ were clumped against a European linkage-disequilibrium reference using a 1 Mb window and *r*^2^ < 0.001. The variance explained by each variant was calculated as $R^{2}=\frac{2\times EAF\times \left(1-EAF\right)\times \beta^{2}}{2\times EAF\times \left(1-EAF\right)\times \beta^{2}+2\times EAF\times \left(1-EAF\right)\times N\times SE^{2}}$, and instrument strength as $F=\frac{R^{2}\times \left(N-2\right)}{1-R^{2}}$, where *β* and SE denote the variant effect and its standard error and *N* denotes sample size. Variants with an individual *F*-statistic ≤ 10 were excluded. The final set contained 5663 variants across 439 microbial traits (Supplementary Table S3). Exposure and outcome alleles were aligned with harmonise_data in TwoSampleMR [32]. Forward-strand alleles were inferred from allele frequencies when possible; incompatible variants and palindromic variants with ambiguous allele frequency were removed. Exposure–outcome pairs with fewer than three harmonized variants were not analyzed. The microbial instruments described above were reused for adult IDPs, childhood volumes, path *a* metabolites, total effects, and AD; metabolite instruments for path *b* were selected separately, as specified below.

For the additional strict-threshold sensitivity analysis, oral microbial instruments were selected at *p* < 5 × 10^−8^ using the same clumping and harmonization rules and the minimum of three usable variants. The two pre-harmonization eligible taxa and all seven variants are listed in Supplementary Table S13, including exclusion of the ambiguous palindromic rs679574. MR was repeated for the one remaining eligible taxon across adult and childhood outcomes. These sensitivity analyses did not relax the minimum instrument count to retain additional taxa.

### 2.4. Mendelian Randomization

Inverse-variance weighted (IVW) MR was the primary estimator. Cochran’s *Q* was calculated for each exposure–outcome pair; a fixed-effect IVW model was used when the IVW *Q*-test *p* ≥ 0.05 and a multiplicative random-effects IVW model otherwise. MR-Egger regression [33], weighted-median [34], weighted-mode, and simple-mode estimators [35] were used as complementary analyses. Directional horizontal pleiotropy was assessed from the MR-Egger intercept. Estimates were reported as *β* values in the units of the source GWAS, with 95% confidence intervals calculated as *β* ± 1.96 × SE. AD effects were exponentiated and reported as odds ratios. The adult screen evaluated 438 microbial traits against 1325 IDPs, the childhood analysis evaluated 434 traits with usable instruments against eight outcomes, and the AD analysis evaluated all 439 traits. Benjamini–Hochberg (BH) correction [36] was applied to the complete family of primary IVW *p* values before the MR-Egger reporting filter: across 438 taxa separately for each adult IDP, across 434 taxa separately for each childhood outcome, and across all 439 taxa for AD. Pairs with MR-Egger intercept *p* < 0.05 were then excluded from primary reporting. Thus, *p* < 0.05 denotes nominal evidence, and BH-adjusted *p* < 0.05 denotes multiplicity-adjusted evidence within the stated family. Raw *p* < 0.001 was used for the adult and childhood overview plots, whereas Figure 6b used raw *p* < 0.01. The 87 adult findings therefore have per-IDP, not atlas-wide, multiplicity support. For the additional *p* < 5 × 10^−8^ sensitivity analysis, which retained only one taxon, BH correction was applied across all 1325 adult primary IVW tests and separately across eight childhood primary IVW tests before the MR-Egger reporting filter.

### 2.5. Two-Step MR Indirect-Effect Analysis

Candidate indirect effects were assessed with a two-step product-of-coefficients framework. For each microbial trait–metabolite–IDP triplet, path *a* was the IVW estimate for microbial trait to metabolite, path *b* was the IVW estimate for metabolite to IDP, and path *c* was the total microbial trait to IDP estimate. The same *Q*-dependent fixed- or random-effects IVW rule and MR-Egger intercept filter were applied to every path. Final candidate paths required raw two-sided *p* < 0.05 for paths *a*, *b*, and *c* and the indirect effect, MR-Egger intercept *p* ≥ 0.05 for all three-component MR analyses, and a mediated proportion > 0 and ≤1. These nominal criteria, rather than a BH-adjusted *p* threshold, defined the 1118 paths carried forward. Microbial instruments were selected as described above. For metabolite exposures in path *b*, instruments were restricted to genome-wide significant variants (*p* < 5 × 10^−8^), clumped against a European linkage-disequilibrium reference using a 1 Mb window and *r*^2^ < 0.001, and required individual *F*-statistics > 10. Exposure–outcome pairs were analyzed only when at least three variants remained after harmonization; no relaxed metabolite instrument threshold was used. The indirect effect was calculated as *a* × *b*. Its standard error was obtained by the delta method, $SE\left(ab\right)=\sqrt{b^{2}\times SE{\left(a\right)}^{2}+a^{2}\times SE{\left(b\right)}^{2}}$, and a two-sided normal-test *p* value was calculated. The approximate direct effect was *c*′ = *c* − *ab*, with $SE\left(c^{\prime }\right)=\sqrt{SE{\left(c\right)}^{2}+SE{\left(ab\right)}^{2}}$, and the mediated proportion was *ab*/*c*. Paths received the descriptive label “partial” when *c*′ remained nominally significant and “complete” otherwise; these labels did not establish biological mediation.

For every reported indirect effect, the 95% CI was calculated as ab ± 1.96 × SE(ab). For the mediated proportion m = ab/c, we additionally propagated uncertainty in the total effect: $SE(m)\approx \sqrt{\frac{{SE(ab)}^{2}}{c^{2}}+\frac{{\left(ab\right)}^{2}{SE(c)}^{2}}{c^{4}}}$, and the approximate 95% CI was m ± 1.96 × SE(m). This is a zero-covariance approximation: joint covariances among a, b, and c were unavailable from the supplied summary results. In particular, shared instruments and outcome datasets may correlate the indirect and total estimates. The intervals are therefore approximate, are not adjusted for selection or multiplicity, and may be unstable when c is small. Proportion limits were not truncated to 0–1. All indirect-effect and mediated-proportion CIs are provided in Supplementary Table S16; dividing the indirect-effect CI by c alone would not account for uncertainty in the denominator.

### 2.6. Imaging GWAS Gene Mapping

The 1325 adult IDP GWAS files were analyzed with MAGMA v1.10 using the default SNP-wise mean gene model [37]. Variants were assigned with the National Center for Biotechnology Information (NCBI) build 37.3 gene-location file, and linkage disequilibrium was estimated from the 1000 Genomes European reference panel. Genes with nominal MAGMA *p* < 0.05 were retained for bulk and spatial gene set scoring. This procedure generated gene results for all 1325 IDPs. Frontal cortex-, cerebellum-, and hippocampus-related IDPs were selected from catalogued trait names and curated IDP descriptions.

### 2.7. Bulk RNA Sequencing

Mouse brain count matrices were obtained from GSE299912 and PRJNA1277304. The dataset contained frontal cortex and cerebellum from ligature-induced periodontitis and control mice, with five samples per condition in each tissue. Deposited gene-count columns were used; precomputed differential-expression columns were ignored. Analyses were performed separately by tissue, and genes were retained when they had at least 10 counts in at least five samples. Tissue-specific variance-stabilized expression matrices generated during preprocessing were used for gene set scoring. Human MAGMA genes were converted to mouse orthologs with babelgene v22.9. For each IDP, all nominal MAGMA genes overlapping the tissue expression matrix were used; gene sets with fewer than five expressed mouse genes were excluded. GSVA scores were calculated from the tissue-specific variance-stabilized expression matrices with a Gaussian kernel [38]. This yielded 154 frontal cortex and 32 cerebellar IDP gene sets. Ligature and control scores were compared with two-sided Wilcoxon rank-sum tests, using exact *p* values when possible and an asymptotic calculation when ties precluded the exact test. The effect was summarized as the difference in group means.

### 2.8. Spatial Transcriptomics

Prefrontal cortex spatial transcriptomic counts were obtained from GSE299911 and PRJNA1277302 (one control section, GSM9049686, and one ligature-induced periodontitis section, GSM9049687). Matrix Market files were restricted to genes shared by both sections and loaded into Seurat [39]. Spots were retained at nFeature ≥ 200, nCount ≥ 500, and mitochondrial transcript percentage ≤ 30%. Log-normalized spatial expression was retained for scoring. SCTransform with mitochondrial percentage regression and 3000 variable genes [40], 50 principal components, a 30-component neighbor graph, and Seurat clustering were used for exploratory spatial organization. Resolution 0.2 clusters were consolidated into six marker- and anatomy-informed domains. The frontal-cortex gene set analysis was restricted to the optimized matched cortical region of interest containing 3858 spots. Within each set, log-normalized expression was z-scored for every gene across spots and averaged by spot; the resulting score was then standardized across all scored spots. Periodontitis and control scores were compared within each of the six domains by two-sided Wilcoxon rank-sum tests, producing 924 tests. BH correction was applied globally across these 924 region–gene set tests. Tests required at least three spots per condition; significance symbols were displayed only for domains with at least 10 spots per condition. With only one section per condition, these spot-level comparisons and adjusted *p* values provide descriptive localization and cannot support inference based on independent biological replicates.

### 2.9. Single-Nucleus RNA Sequencing

Hippocampal single-nucleus RNA-seq data were obtained from CRA007555 (BioProject PRJCA010526), deposited by Shen et al. [41]. The analyzed dataset comprised four PD and four PDT libraries and 46,547 nuclei annotated as neurons, astrocytes, oligodendrocytes, oligodendrocyte precursor cells, microglia, endothelial cells, or pericytes. In total, 50 hippocampal IDP gene sets were scored on log-normalized expression using the GSVA implementation of ssGSEA [38]. For the primary analysis, nucleus-level scores were averaged within each library and cell type. A library–cell-type combination required at least three nuclei, and each comparison required four libraries per condition. Unpaired two-sided Wilcoxon rank-sum tests compared the four PDT and four PD library means using the continuity-corrected normal approximation (exact = FALSE). Effects were summarized as differences between the means of library-level scores. BH adjustment was applied globally across all 350 cell-type–gene set tests; within-cell type (50 tests) and within-gene set (seven cell types), adjustments are also provided in Supplementary Table S21.

For the retained exploratory nucleus-level displays, PD and PDT scores were compared within cell type by two-sided Wilcoxon rank-sum tests with at least 20 nuclei per group, followed by global BH adjustment across 350 comparisons. Supplementary Table S22 reports PDT-minus-PD nucleus-level mean differences; the volcano plots use log_2_(mean score in PDT/mean score in PD). Correlated nuclei do not constitute independent biological replicates, so these *p* values are descriptive and not used for population-level inference.

### 2.10. Full-Length 16S Ribosomal RNA Profiling

Full-length oral 16S ribosomal RNA Oxford Nanopore reads were obtained from BioProject PRJNA1243403, generated in the AD–life partner study [42]. The 101 biospecimens represented 51 participants: 50 saliva samples (25 AD, 25 controls) and 51 supragingival-plaque samples (26 AD, 25 controls). Patient26 contributed plaque only. The source report states that Patient02 participated without a partner, and Patient26 could not provide sufficient saliva. The saliva set contained 24 complete household pairs plus unmatched Patient02 and Control26; the plaque set contained 25 complete pairs plus unmatched Patient02. Thus, 50 participants belonged to 25 recruited patient–partner households, with one additional unpaired patient. Abundance comparisons were unpaired and used all available samples for the corresponding site. FASTQ files were profiled with Emu v3.4.5 [43] using minimap2 v2.30 [44] and the Emu reference database. Emu abundance was run with estimated counts retained, four threads, and a minimap2 frequency threshold of 0.005. Unassigned reads were excluded. Species-level relative abundances were obtained by summing identical taxonomic lineages and filling absent taxa with zero. Of the 35 nominally AD-associated MR taxa, 15 exact species were represented. Two-sided Mann–Whitney *U* tests compared abundance between groups within each site. Three saliva taxa with raw *p* < 0.05—*Selenomonas infelix*, *Capnocytophaga granulosa*, and *Dialister pneumosintes*—were carried forward. Their selection in the evaluation cohort makes all subsequent classification estimates exploratory.

### 2.11. Exploratory Classification Modeling

Single-taxon and summed-abundance discrimination was quantified by ROC analysis. The summed score was the unweighted sum of the three raw abundance percentages; these were in-cohort fixed-score estimates. Logistic models used the three taxa as separate predictors. Numeric predictors were median-imputed and standardized within each training fold; categorical predictors were most-frequent imputed and one-hot encoded within training folds. Logistic regression used L2 regularization (*C* = 1), the liblinear solver, balanced class weights, and a maximum of 5000 iterations. The nine clinical covariates eligible for subset selection were age, weight, height, body mass index, education years, number of teeth, sulcus bleeding index, sex, and smoking status. MMSE was excluded a priori.

Clinical-covariate selection was nested within outer LOOCV. In each of 50 outer folds, one participant was reserved for testing. Within the other 49 participants, all 512 subsets of the nine covariates, each added to the fixed three taxa, were compared by fivefold stratified inner cross-validation. Selection maximized mean inner AUC; ties favored fewer clinical variables and then lexical feature order. The selected pipeline was refitted on the full outer training set and used once to predict the held-out participant. The 50 out-of-fold probabilities defined the primary AUC. Percentile 95% CIs used 10,000 class-stratified bootstrap resamples of these fixed predictions. All preprocessing was refitted within the relevant training fold. Supplementary Table S24 reports the inner-AUC distribution and exact-subset selection frequency across the 50 outer folds.

A household-grouped nested sensitivity analysis used leave-one-household-out outer validation and fivefold stratified group cross-validation for inner subset selection. Both members of each complete saliva pair were kept together; unmatched Patient02 and Control26 each formed a one-member group, giving 26 outer folds. Models were refitted on the remaining groups and predicted only the held-out household. Uncertainty used 10,000 household-cluster bootstrap replicates, and Figure 6g summarizes selection stability. This sensitivity analysis was separate from the historical site refits in Figure 6h. The non-nested education-augmented model and the algorithm comparisons are secondary post-selection analyses.

XGBoost [45] was compared with logistic regression for the three-taxon and three-taxon-plus-education feature sets. Hyperparameters were tuned with Optuna’s tree-structured Parzen estimator [46] within each outer LOOCV training set using fivefold stratified inner cross-validation and 15 trials. Search ranges were 20–280 trees, depth 1–4, learning rate 0.005–0.20, subsampling and feature sampling 0.50–1.00, minimum child weight 0.5–12, *γ* 0–5, L1 penalty 10^−4^–10, L2 penalty 0.1–50, and maximum delta step 0–5. Algorithm comparisons used 10,000 paired class-stratified bootstrap replicates of fixed out-of-fold predictions; model fitting and tuning were not repeated within replicates. The education-augmented comparison remains secondary and post-selection because education was chosen using the same cohort. For the historical site comparison in Figure 6h, the fixed three-taxon-plus-education logistic pipeline was refitted and evaluated separately in all available saliva (n = 50) and plaque samples (n = 51). Its exploratory AUC difference used 5000 independent class-stratified bootstrap replicates of fixed site-specific predictions. This calculation does not account for shared participants or household dependence and is not a matched-site or external-transfer test.

### 2.12. Statistical Analysis and Software

Unless otherwise specified, tests were two-sided. No observations were removed as statistical outliers. Missing values were handled only by the analysis-specific procedures described above. MR and transcriptomic analyses used R 4.3.2 with TwoSampleMR 0.6.2, MAGMA 1.10, GSVA 1.50.5, Seurat 5.0.1, babelgene 22.9, data.table 1.17.8, and matrixStats 1.2.0. Microbiome and prediction analyses used Python 3.9.13 with pandas 2.3.3, NumPy 2.0.2, SciPy 1.13.1, scikit-learn 1.6.1 [47], XGBoost 2.1.4, and Optuna 4.9.0. Random seeds were fixed in all stochastic workflows.

## 3. Results

### 3.1. A Genetically Informed Atlas Reveals Anatomically Specific Oral Microbiome–Brain Associations

The overall analytical framework is summarized in Figure 1. The oral microbiome GWAS provided 5663 single-nucleotide polymorphism (SNP)–taxon instruments for 439 species-level traits, with 3–26 instruments per taxon (median, 13). All retained instruments exceeded the prespecified F-statistic threshold (minimum *F*-statistic, 20.9; median, 22.3), and 438 taxa yielded complete harmonized estimates for the adult brain analysis (Supplementary Tables S1–S3). These exposures were tested against 1325 structural IDPs assigned to eight anatomical classes, generating 580,350 primary inverse-variance weighted (IVW) estimates. The primary atlas was complemented by MR-Egger, weighted-median, weighted-mode, and simple-mode estimates, together with Cochran’s *Q* and MR-Egger intercept tests (Supplementary Tables S4–S11).

To distinguish the strongest signals from this broad nominal landscape, Benjamini–Hochberg (BH) correction was applied across the 438 taxa tested for each IDP. In total, 87 associations met this within-IDP BH threshold (BH-adjusted *p* < 0.05), spanning 51 taxa and 79 IDPs; 42 coefficients were positive, and 45 were negative. These signals were anatomically distributed, with 17 in the occipital/visual class, 16 in parietal/cingulate, 15 in frontal, 14 in temporal/insula, 12 in subcortical/limbic, 5 each in sensorimotor and cerebellum/brainstem, and 3 in the global/white matter/other class. Directional pleiotropy was uncommon among the most strongly supported pairs: only 8 of the 587 raw associations with *p* < 0.001 had an MR-Egger intercept *p* < 0.05, and only one of the retained *p* < 0.001 associations showed Cochran’s *Q* evidence of heterogeneity. The BH correction is specific to each IDP’s family of taxa and does not control the false discovery rate across the full atlas of 580,350 tests.

After excluding pairs with evidence of directional horizontal pleiotropy (MR-Egger intercept *p* < 0.05), 28,178 taxon–IDP associations met the nominal *p* < 0.05 threshold, including 5925 at *p* < 0.01 and 579 at *p* < 0.001 (Figure 2a,b). Effect directions were balanced, with 13,442 positive and 14,736 negative coefficients. The largest numbers of nominal associations involved temporal/insula IDPs (5787), frontal IDPs (4927), occipital/visual IDPs (4493), and parietal/cingulate IDPs (4286), followed by subcortical/limbic (3675), sensorimotor (3015), global/white matter/other (1085), and cerebellum/brainstem traits (910). At the microbial level, Firmicutes contributed 9278 nominal pairs, followed by Bacteroidetes (5918), Proteobacteria (3706), and Actinobacteria (3568), with additional associations distributed across nine less abundant phyla (Figure 2b).

The leading association retained after the MR-Egger intercept filter linked genetically proxied *Campylobacter* SGB19298 abundance to lower left ventral-lateral-anterior thalamic volume (*β* = −0.297, 95% confidence interval (CI) −0.416 to −0.177, *p* = 1.20 × 10^−6^, BH-adjusted *p* = 5.24 × 10^−4^; Figure 2a,c). *Streptococcus rubneri* was associated with both greater right subparietal sulcal surface area (*β* = 0.523, 95% CI 0.302–0.744, *p* = 3.42 × 10^−6^) and greater volume of the same parcel (*β* = 0.521, 95% CI 0.300–0.741, *p* = 3.83 × 10^−6^). GGB2672 SGB3600 was associated with greater left cuneus area in two parcellation schemes, including the Desikan–Killiany–Tourville (DKT) atlas estimate (*β* = 0.431, 95% CI 0.249–0.614, *p* = 3.62 × 10^−6^), whereas *Dialister micraerophilus* was associated with greater left caudate volume (*β* = 0.330, 95% CI 0.188–0.473, *p* = 5.60 × 10^−6^). These leading signals remained directionally concordant across the complementary MR estimators (Supplementary Tables S4–S11). The selected heat map emphasized that individual taxa frequently mapped to more than one anatomically related phenotype, whereas the metric-stratified views showed distinct associations with cortical thickness, surface area, and volume (Figure 2c and Figure S1). Thus, the adult analysis did not resolve to a single global brain-size axis, but instead nominated region- and metric-specific taxon–structure relationships.

### 3.2. Pediatric Analyses Identify Suggestive Oral Microbiome–Brain Associations

We next tested whether genetically proxied oral microbial abundance was related to brain structure earlier in life. Valid harmonized estimates were available for 434 taxa and 8 childhood volumetric outcomes, yielding 3472 taxon–outcome pairs and 17,360 estimates across 5 MR methods (Figure 3 and Supplementary Table S12). In total, 53 pairs showed an MR-Egger intercept *p* < 0.05 and were treated as potentially pleiotropic. Among the remaining pairs, 196 IVW associations met *p* < 0.05, 41 met *p* < 0.01, and 1 met *p* < 0.001; 146 taxa had at least one nominally supported childhood association. The number of *p* < 0.05 signals ranged from 20 for hippocampal volume to 31 for pallidal volume, indicating that the nominal signal was distributed across all eight structures rather than being concentrated in one outcome. The strongest association linked *Peptostreptococcus stomatis* to greater intracranial volume (*β* = 1.009, *p* = 3.01 × 10^−4^). Other leading estimates included genetically proxied *Fusobacterium nucleatum* abundance with lower putamen volume (*β* = −1.357, *p* = 0.00153), *Gemella* SGB7284 with lower intracranial volume (*β* = −0.577, *p* = 0.00154), *Veillonella* sp. 3627 with greater intracranial volume (*β* = 0.826, *p* = 0.00163), and *Lachnoanaerobaculum orale* with lower pallidal volume (*β* = −0.910, *p* = 0.00168). No childhood association survived BH correction across taxa within its corresponding outcome. These nominal findings are suggestive and exploratory.

We additionally repeated the adult and childhood analyses using genome-wide significant oral microbial instruments (*p* < 5 × 10^−8^). Only GGB6646 SGB9384 (four instruments) and *Granulicatella* SGB8239 (three instruments) met the pre-harmonization minimum. The palindromic SNP rs679574 was excluded because its allele frequency was ambiguous, leaving *Granulicatella* SGB8239 with two usable variants and therefore ineligible for MR (Supplementary Table S13). GGB6646 SGB9384 retained four variants for 1325 adult IDPs and eight childhood outcomes. The adult analysis yielded 38 nominal IVW associations (minimum *p* = 0.00223); none survived BH correction across the 1325 outcomes (minimum adjusted *p* = 0.998; Supplementary Table S14). No childhood IVW association reached *p* < 0.05 (minimum *p* = 0.0590 for thalamic volume; minimum BH-adjusted *p* = 0.472 across eight outcomes; Supplementary Table S15). These restricted sensitivity results do not confirm the robustness of the leading atlas findings.

### 3.3. Exploratory Metabolomic Integration Nominates Candidate Routes Linking Oral Taxa to Brain Structure

To identify circulating intermediates that could connect oral microbial traits to brain structure, we performed a two-step MR across 1400 plasma metabolite traits. Applying the prespecified nominal inclusion rules—raw two-sided *p* < 0.05 for the taxon-to-metabolite, metabolite-to-IDP, total and indirect effects; MR-Egger intercept *p* ≥ 0.05 for all three component MR analyses; and a mediated proportion >0 and ≤1—yielded 1118 exploratory candidate indirect-effect paths (Figure 4 and Figure S2 and Supplementary Table S16). These paths connected 261 oral taxa, 253 metabolites, and 605 adult IDPs. They were most numerous for temporal/insula outcomes (*n* = 220) and subcortical/limbic outcomes (*n* = 201), followed by occipital/visual (*n* = 188), parietal/cingulate (*n* = 138), frontal (*n* = 137), cerebellum/brainstem (*n* = 109), sensorimotor (*n* = 91), and global/white matter/other traits (*n* = 34). Under the prespecified residual-direct-effect rule, the residual direct effect did not reach nominal significance in 729 paths and remained nominally significant in 389. These descriptive labels do not establish complete or partial biological mediation. The nominal screening criteria, rather than multiplicity-adjusted significance, defined these paths, which should be interpreted as hypothesis-generating.

The strongest nominal indirect-effect association linked *Prevotella veroralis* through succinylcarnitine (C4DC) to left thalamic volume (indirect *β* = 0.0422, *p* = 8.21 × 10^−4^; mediated proportion, 30.8%). A related *P. veroralis*–C4DC path involved right mediodorsal-lateral thalamic volume (indirect *β* = 0.0293, *p* = 0.00482; mediated proportion, 16.6%; Figure 4d). Other examples were *Porphyromonas gingivalis* through 1-methylhistidine to lower left thalamic volume (indirect *β* = −0.0631, *p* = 0.0482; 22.3% mediated; Figure 4b) and *Fusobacterium nucleatum* through the hypotaurine-to-taurine ratio to lower left insular surface area (indirect *β* = −0.1046, *p* = 0.0365; 29.2% mediated; Figure 4c). The GGB45697 SGB63414–guanidinoacetate path involved greater right amygdala volume (indirect *β* = 0.1405, *p* = 0.00874; 39.7% mediated; Figure 4e), whereas the *Streptococcus vestibularis*–1-linoleoyl-2-arachidonoyl-glycerophosphocholine path involved lower right dentate-gyrus head volume (indirect *β* = −0.0266, *p* = 0.0367; 14.7% mediated; Figure 4f). Thus, 95% confidence intervals for all reported indirect effects and mediated proportions, calculated using delta method standard errors, are provided in Supplementary Table S16. Additional pathways are shown in Supplementary Figure S2.

### 3.4. Exploratory Transcriptomic Analyses Localize Candidate Brain-Structure Programs

We next asked whether experimental periodontitis altered transcriptional programs derived from brain-structure GWAS. MAGMA-derived gene sets for 154 frontal IDPs and 32 cerebellar IDPs were evaluated by gene set variation analysis (GSVA) in bulk RNA-sequencing data from control and ligature-induced periodontitis mice (*n* = 5 per group for each tissue; Supplementary Tables S17–S19). Nine frontal gene sets and one cerebellar gene set differed at nominal *p* < 0.05 (Figure 5a). In the frontal cortex, the gene sets corresponding to right frontomarginal volume and area increased in periodontitis, whereas seven gene sets—including left superior-frontal, middle-frontal, and right inferior-frontal measures—showed lower scores. In the cerebellum, the left lobules I–IV gene set increased.

Spatial transcriptomics provided substantially greater anatomical resolution. A total of 3858 spots from anatomically matched cortical regions of interest were included in gene set testing, comprising 1922 spots from the control section and 1936 from the periodontitis section (Figure 5b and Supplementary Table S20). Across 154 frontal IDP gene sets and six regions, 346 of 924 exploratory spot-level comparisons had global Benjamini–Hochberg (BH)-adjusted *p* < 0.05, including 154 at BH-adjusted *p* < 0.01. The direction of change differed sharply by region: region 1 contained 103 gene sets meeting the spot-level threshold (72 higher and 31 lower in periodontitis); region 2 contained 24 (14 higher and 10 lower); regions 4 and 5 contained 63 and 84, respectively, all lower, whereas all 72 gene sets meeting the spot-level threshold in region 6 were higher. Region 3, represented by only 33 control and four periodontitis spots, had no BH-supported gene sets. Because each condition was represented by one section, these spot-level results localize candidate responses but do not provide replicate-aware biological inference. Condition differences are inseparable from animal, section, and technical variation in this design.

We evaluated hippocampal single-nucleus RNA sequencing from four periodontitis (PD) and four periodontitis-after-treatment (PDT) libraries comprising 17,499 and 29,048 nuclei, respectively (Figure 5d). In total, 50 IDP gene sets were scored in seven cell types. Because nuclei from the same library are correlated, primary comparisons used the mean ssGSEA score within each library and cell type, giving four PD and four PDT values for each of 350 tests (Supplementary Table S21). Only the right CA3-head volume gene set in microglia reached nominal significance (PDT-minus-PD mean difference, −0.0326; two-sided Wilcoxon *p* = 0.0304; within-cell-type BH-adjusted *p* = 0.803; global BH-adjusted *p* = 1.000). No comparison survived the BH correction. This library-level analysis therefore provides no multiplicity-adjusted evidence of a treatment-associated gene set difference.

The nucleus-level analyses are retained as an exploratory view of within-library variation (Figure 5e–l and Supplementary Table S22). They yielded 233 of 350 comparisons with global BH-adjusted *p* < 0.05, including 48 in oligodendrocytes, 47 each in microglia and oligodendrocyte precursor cells, 46 in astrocytes, 36 in neurons, 8 in pericytes, and 1 in endothelial cells. The extreme right CA3-body neuronal result (*p* = 6.71 × 10^−72^; BH-adjusted *p* = 2.35 × 10^−69^) arises from treating correlated nuclei as independent observations. These *p* values and counts are descriptive and must not be interpreted as valid population-level evidence or as a substitute for the library-level results.

### 3.5. Genetic Prioritization and Salivary Profiles Inform an Exploratory AD Classifier

We finally tested whether oral taxa linked genetically to brain structure also showed evidence of association with AD and classification performance in human oral microbiome data. Among 439 taxon–AD MR analyses, 35 taxa had nominal IVW *p* < 0.05 and MR-Egger intercept *p* ≥ 0.05; 18 were associated with higher AD odds and 17 with lower odds (Figure 6a and Supplementary Table S23). In total, 10 associations had *p* < 0.01, and two had *p* < 0.001. One taxon, GGB12443 SGB19313, survived Benjamini–Hochberg correction across all 439 taxa (odds ratio (OR) = 1.378, 95% CI 1.186–1.601, *p* = 2.77 × 10^−5^, BH-adjusted *p* = 0.0122). At the raw IVW *p* < 0.01 threshold used in Figure 6b, the 35 AD-linked taxa participated in 468 adult brain associations retained after the MR-Egger intercept filter involving 384 distinct IDPs: 233 regional or tissue-volume, 164 surface-area, and 71 cortical-thickness associations. These pairs spanned all eight anatomical classes and were directionally balanced (249 positive and 219 negative).

Of the 35 nominally AD-associated taxa, 15 exact species were present in the full-length 16S matrix. Three were prioritized because their salivary abundance differed between controls and participants with AD at raw two-sided Mann–Whitney *U* test, *p* < 0.05. Genetically proxied *Selenomonas infelix* abundance was associated with higher AD odds (OR = 1.110, 95% CI 1.004–1.228, *p* = 0.0407), whereas *Capnocytophaga granulosa* (OR = 0.886, 95% CI 0.800–0.981, *p* = 0.0199) and *Dialister pneumosintes* (OR = 0.852, 95% CI 0.726–1.000, *p* = 0.0496) showed protective-direction MR estimates. All three taxa were more abundant in AD saliva (*S. infelix*, *p* = 0.00575; *C. granulosa*, *p* = 0.0145; *D. pneumosintes*, *p* = 0.0324; Figure 6c).

As in-cohort fixed abundance scores, the three taxa yielded AUCs of 0.725, 0.698, and 0.670, respectively (Figure 6d). Their summed abundance was higher in AD (*p* = 4.37 × 10^−4^) and yielded a fixed-score AUC of 0.790 (Figure 6e). The fixed three-taxon logistic model achieved a LOOCV AUC of 0.714 (bootstrap 95% CI, 0.550–0.861). To address optimism from clinical-variable selection, all 512 subsets of nine clinical covariates were compared within each outer training set, and the selected pipeline predicted only the held-out participant. Pooled out-of-fold predictions yielded the primary nested AUC of 0.784 (95% CI, 0.646–0.901; Figure 6f). Supplementary Table S24 summarizes all 512 subsets’ inner-validation performance and selection frequencies.

Education was selected in all 50 individual outer folds, followed by age in 16 and smoking status in 11 (Figure 6g). With household-grouped nested validation, education was selected in 25 of 26 outer folds, and the pooled AUC was 0.810 (household-cluster bootstrap 95% CI, 0.678–0.926). The original non-nested three-taxon-plus-education AUC of 0.843 is retained only as a secondary, optimistic post-selection benchmark (Supplementary Figure S3b); it is not an unbiased estimate of the selection procedure. Logistic regression and nested-tuned XGBoost yielded AUCs of 0.714 and 0.667 for the fixed three-taxon set and 0.843 and 0.739 after adding education (Supplementary Figure S3). For the historical same-site refits, the education-augmented model yielded an AUC of 0.843 in saliva (95% CI, 0.725–0.939) and 0.542 in plaque (95% CI, 0.378–0.695; Figure 6h). This last comparison uses unequal specimen sets and independent bootstrap resampling and is descriptive; it does not establish performance differences in matched participants. All models remain exploratory proof-of-concept classifiers requiring independent external validation.

## 4. Discussion

This study provides a genetically anchored map of species-level oral microbial associations with anatomically resolved brain phenotypes. In total, 87 taxon–IDP associations passed BH correction across taxa within individual IDPs; they are not discoveries under atlas-wide correction across all 580,350 tests. MR can reduce susceptibility to reverse causation and shared environmental confounding under its instrumental-variable assumptions, but does not by itself establish a temporally specific or mechanistically resolved causal pathway [14]. Here, the instruments proxy a heritable propensity toward relative taxon abundance and originate from one autism-ascertained family cohort whose exposure effects have not been externally validated in the outcome populations. These features connect the directionality motivation in the Introduction to our cautious interpretation of the atlas. The childhood, metabolomic, transcriptomic, and clinical modules add exploratory hypotheses for subsequent testing.

The adult atlas replaces a composite diagnosis of periodontitis with taxon-resolved exposures and surveys distinct morphometric properties across the brain. This species-level view complements recent multiscale MR work spanning salivary and gut microbial traits [18], while providing a substantially denser oral catalogue and broader structural phenome. Leading associations involved thalamic nuclei, caudate, cuneus, and subparietal cortex rather than converging on global brain size. This pattern is consistent with population imaging studies linking periodontal status to white matter, hippocampal, and cortical phenotypes [6,7,8,9] and with oral microbiome studies reporting associations with cortical and subcortical measures [10,11]. It also resembles gut microbiome–imaging work in which relationships depend on the anatomical and imaging phenotype considered [16,17]. Cortical thickness and surface area have partly distinct genetic architectures [48], making their separation biologically informative. The near balance of positive and negative estimates likewise argues against a model in which every putatively adverse organism drives uniform tissue loss. Individual taxa may instead index distinct ecological functions, inflammatory states, or host responses whose brain correlates depend on community and anatomical context. MR here proxies a heritable propensity toward relative taxon abundance [14,20,21], which does not identify a specific exposure window or establish an effect of acute infection.

The childhood and metabolite screens extend the anatomical map across developmental and systemic dimensions, but remain suggestive and exploratory. No childhood association survived outcome-specific BH correction, and the metabolite paths were selected by nominal criteria without multiplicity-controlled support. Brain structure follows nonlinear, region-specific trajectories across the lifespan [24,49], making age-specific replication important. Candidate metabolites include amino-acid derivatives, acylcarnitines, taurine-related measures, and phospholipids represented in the source plasma-metabolome resource [30]. Recurrent thalamic and insular paths nominate targets for repeated-sampling and targeted metabolomic studies. The broad approximate CIs for mediated proportions further caution against interpreting these statistical decompositions as established in vivo mediation.

The transcriptomic analyses describe tissue and cellular settings in which periodontal perturbation may intersect with brain-structure gene programs. Bulk-tissue differences and spatial patterns nominate regional responses, while nucleus-level profiles describe variation across glial, neuronal, and vascular cell classes. These lineages participate in immune surveillance, myelin maintenance, and neurovascular function [50,51,52], and experimental studies have linked periodontitis to cerebral immune activation, blood–brain barrier permeability, and hippocampal responses [41,53,54]. However, our library-level reanalysis yielded only one nominal microglial association and no BH-supported difference. Accordingly, neither the extreme nucleus-level *p* values nor the one-section-per-condition spatial comparisons establish replicated population-level effects. They provide candidate locations for independently replicated experiments.

The genetic and clinical directions require separate interpretation. Independent cohorts report altered oral communities in AD, with differences across disease stages and oral niches [42,55,56]. In this study, *Selenomonas infelix* showed concordant MR and salivary directions, whereas *Capnocytophaga granulosa* and *Dialister pneumosintes* were enriched in AD saliva despite protective-direction MR estimates. The MR exposure is a host-genetic propensity toward normalized relative abundance across the source population; the clinical measure is abundance after AD has developed. These are different exposure contrasts and time windows. Disease-related changes in diet, oral self-care, dentition, salivary physiology, and medication use could therefore enrich a species without implying that lifelong exposure to it increases AD risk. The source clinical study specifically discusses medication-related microbiome confounding [42].

Measurement and biological context offer additional possible explanations. Relative abundance is compositional: an apparent increase may reflect depletion of other organisms rather than increased absolute microbial load. Metagenomic species bins and full-length 16S profiles also do not capture identical strain-level or functional variation, and a species may have different ecological effects across communities. Finally, the two protective-direction MR estimates are only nominally significant, leaving sampling error, horizontal pleiotropy, and imperfect transportability as alternatives to true protection. A related distinction appears in the broader literature: longitudinal periodontal associations with dementia [5] have not been consistently reproduced by genetic analyses. Sun et al. found a nominal periodontitis–AD association with one instrument set that was not supported by an independent set [57]. That evidence concerns periodontitis as a clinical exposure and does not resolve the directions of the individual taxa here. Thus, salivary enrichment should not be treated as directional confirmation of MR, and neither enrichment nor an OR below 1 establishes a harmful or protective mechanism.

The classifier also remains a within-cohort hypothesis. Its primary nested AUC was 0.784. Education was consistently selected, but its association may reflect cognitive reserve and correlated social or health factors [58,59] as well as the life-partner recruitment design. Household-grouped validation addresses some dependence between partners; it does not make the sample population representative. The education contribution and the site-specific estimates, therefore, require evaluation in independently recruited cohorts with population controls.

A major strength is the deliberate division of labor across data types. Genetic analyses provided directionally oriented, anatomically resolved prioritization; developmental and metabolite screens extended the temporal and systemic scope; transcriptomic data localized candidate tissue responses; and oral sequencing tested disease-state discrimination. Species-level instruments, 1325 adult IDPs, separate adult and childhood outcomes, and multiple transcriptomic resolutions provide greater biological resolution than analyses of periodontitis as a single exposure. This architecture distinguishes a primary adult genetic map from hypotheses for experimental or clinical testing.

Instrument selection and ancestry limit inference. Microbial instruments were selected at *p* < 5 × 10^−6^; exceeding the F-statistic threshold does not establish instrument validity or eliminate weak-instrument bias and winner’s curse. At *p* < 5 × 10^−8^, only one taxon remained analyzable after harmonization, and neither the adult nor childhood primary results survived outcome-family BH correction. This restricted sensitivity analysis cannot validate the leading atlas taxa or the atlas as a whole. Most outcome GWAS participants were of European ancestry, and LD clumping used a European reference. Differences in allele frequencies, LD, oral ecology, and environmental exposures limit extension to non-European populations.

SPARK ascertainment and transportability are separate limitations. The exposure GWAS arose from an autism-ascertained family cohort, in which affected/unaffected family composition, household-correlated diet, and medication may alter genotype–microbiome relationships. *F*-statistics and MR-Egger diagnostics do not test whether those relationships generalize to UK Biobank, the pediatric cohort, or the AD GWAS. The source GWAS reported that its leading microbiome associations were not driven by autism status [21], but this within-cohort observation is not external replication. We did not compare SNP–taxon effect sizes or allele frequencies against an independent oral GWAS. The 610-participant Danish saliva GWAS used a 16S-based, predominantly higher-taxonomic panel rather than a directly comparable species-resolved panel [19]; the Shenzhen metagenomic oral GWAS provides species-level data from Chinese participants [20], with differences in ancestry, geography, and microbial definitions. A comparable population-based European-ancestry species-level replication was not available for this analysis. The transportability assumption remains untested and requires independent, harmonizable GWAS resources.

Multiplicity and biological replication further constrain interpretation. The secondary screens were not restricted to the 87 adult associations supported by within-IDP BH correction. The spatial dataset contains one control and one periodontitis section and cannot separate condition effects from animal, section, or technical variation. Thousands of spots do not supply independent biological replicates, and BH adjustment cannot remedy that design. The single-nucleus primary tests use eight library-level values and yielded no BH-supported difference. Larger experiments with independent animals and sections are needed before interpreting the regional or cell-type patterns biologically.

Clinical generalizability is limited by the small cohort, in-cohort taxon selection, and life-partner controls. Nesting all 512 clinical subsets removes the additional reuse of test data for that selection step, but not the earlier selection of the three taxa. The household sensitivity analysis used only 26 groups. Cohabiting controls share diet, oral hygiene, and socioeconomic conditions with cases, while assortative pairing may correlate with educational attainment within households. These features specifically limit transfer of the education-augmented classifier to population controls, despite the selection stability in Figure 6g. Bootstrap CIs based on fixed out-of-fold predictions do not incorporate the uncertainty of rerunning the complete model-development process. Independent external cohorts, followed by prospective assessment of calibration and clinical utility, are required.

## 5. Conclusions

This study prioritizes oral microbial taxa and adult brain phenotypes for further investigation using a genetically anchored association atlas. Childhood, metabolomic, and transcriptomic findings remain exploratory, and the salivary classifier is an internally evaluated proof of concept. Independent species-resolved GWAS, replicated animal experiments, and external clinical cohorts are needed to test the proposed associations and their potential relevance to AD.

## Figures and Tables

**Figure 1:**
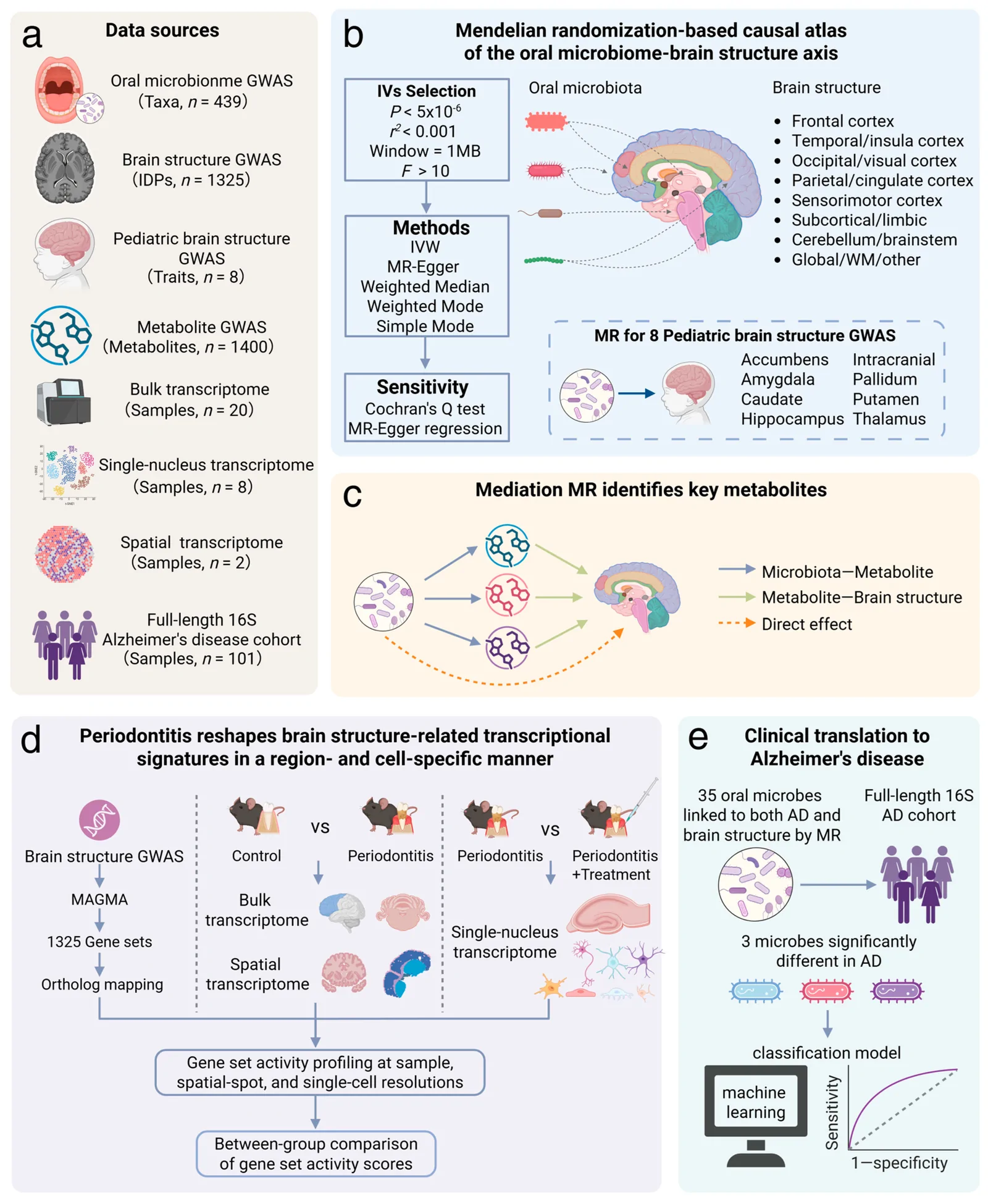
**Study design for cross-scale analysis of the oral microbiome and brain structure.** (**a**) Data sources comprised genome-wide association study (GWAS) summary statistics for 439 species-level oral microbial traits, 1325 adult structural imaging-derived phenotypes (IDPs), eight childhood brain-volume traits, 1400 plasma metabolite traits, and Alzheimer’s disease (AD), together with mouse bulk RNA sequencing (20 libraries), spatial transcriptomics (two sections), hippocampal single-nucleus RNA sequencing (eight libraries; 46,547 nuclei), and full-length oral 16S ribosomal RNA sequencing (101 saliva or supragingival-plaque biospecimens from 51 participants). (**b**) Adult and childhood two-sample Mendelian randomization (MR) workflow. Instruments were selected at *p* < 5 × 10^−6^, linkage-disequilibrium pruned at *r*^2^ < 0.001 within 1 Mb and required *F* > 10. Inverse-variance weighted (IVW) MR was the primary estimator; MR-Egger, weighted median, simple mode, and weighted mode provided sensitivity estimates. Cochran’s *Q* assessed heterogeneity, and the MR-Egger intercept assessed directional horizontal pleiotropy. Adult IDPs were grouped into eight anatomical regions. (**c**) Two-step MR indirect-effect analysis tested oral taxon→metabolite (path *a*), metabolite→IDP (path *b*), oral taxon → IDP total effect, and the delta method indirect effect. Metabolite instruments for path *b* were restricted to *p* < 5 × 10^−8^. (**d**) IDP GWAS signals were converted to gene sets by Multi-marker Analysis of GenoMic Annotation (MAGMA), mapped to mouse orthologs, and scored in bulk, spatial, and single-nucleus transcriptomes to evaluate regional and cell-type dependence under periodontitis and treatment. (**e**) In total, 35 nominally AD-associated oral taxa were cross-referenced with adult IDPs; taxa with nominal between-group differences in the cohort were evaluated as exploratory salivary classification features using logistic regression and XGBoost. Arrows denote analytical flow rather than established biological causation. Of the 439 source taxa, 438 yielded adult estimates. The strict-threshold MR sensitivity analyses, library-level single-nucleus testing, and nested clinical-covariate selection are described in Methods. Childhood and metabolite screens and the clinical classifier are exploratory.

**Figure 2:**
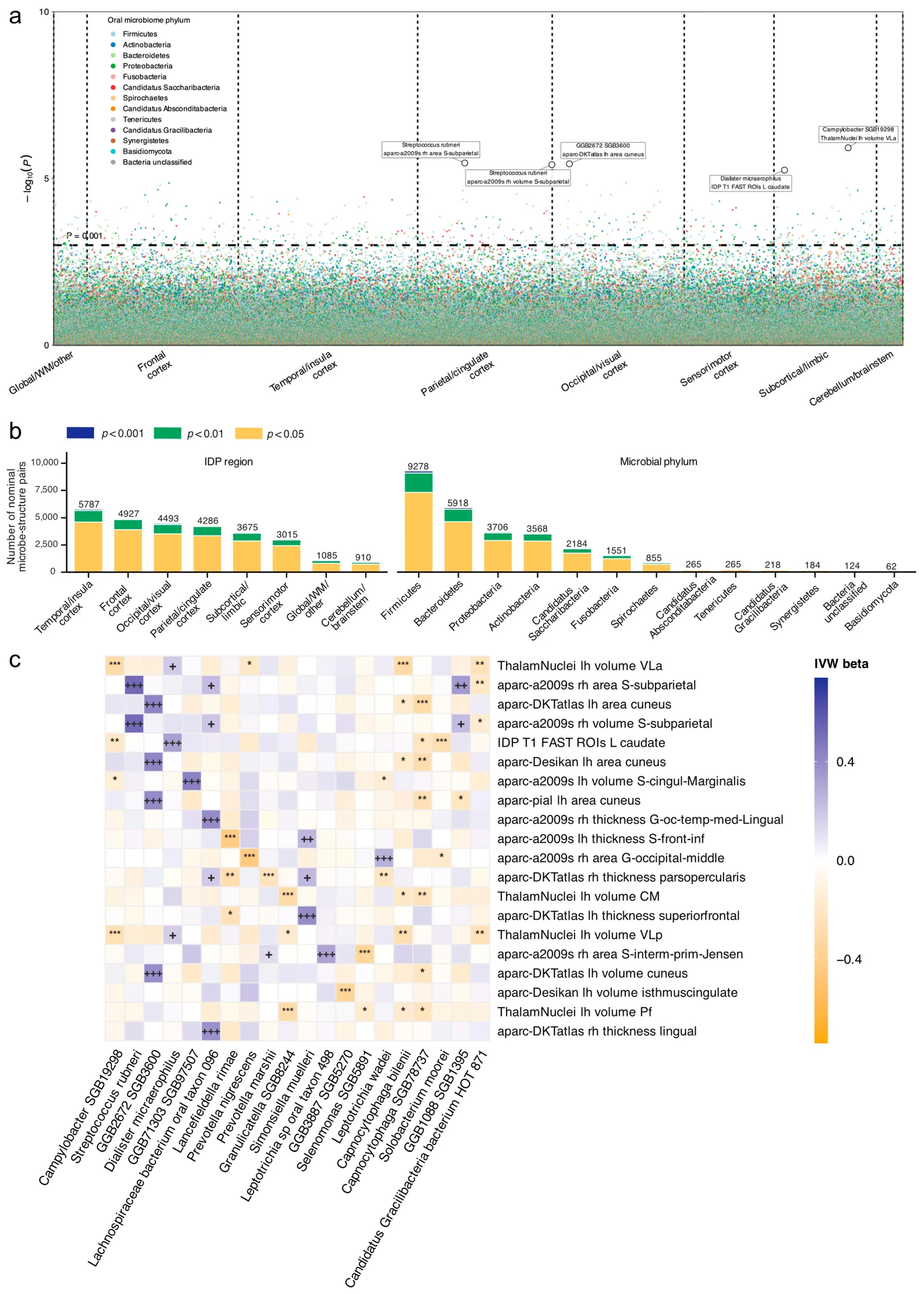
**A genetically informed atlas of oral microbial associations with adult brain structure.** (**a**) Manhattan-style display of 580,350 primary inverse-variance weighted (IVW) Mendelian randomization (MR) estimates generated from 438 oral microbial traits and 1325 adult brain imaging-derived phenotypes (IDPs). Points show −log_10_(two-sided *p*) and are grouped by eight anatomical classes; point color denotes microbial phylum. The horizontal dashed line marks *p* = 0.001, and labels identify selected leading associations without evidence of directional horizontal pleiotropy. Fixed-effect or multiplicative random-effects IVW estimates were used according to between-instrument heterogeneity. (**b**) Number of nominally supported microbe–IDP pairs after excluding associations with an MR-Egger intercept *p* < 0.05. Separate bar charts summarize counts by brain region and by microbial phylum. The stacked colors partition nominal associations into 0.01 ≤ *p* < 0.05, 0.001 ≤ *p* < 0.01, and *p* < 0.001 bins. Counts refer to nominal microbe–IDP pairs, irrespective of the sign of the IVW coefficient. (**c**) Heat map of selected associations between 20 oral taxa and 20 IDPs. Cell color represents the IVW coefficient (blue, positive; orange, negative). For positive coefficients, +, ++, and +++ denote *p* < 0.05, *p* < 0.01, and *p* < 0.001, respectively; for negative coefficients, *, **, and *** denote the same thresholds. Symbols represent unadjusted two-sided *p* values. Within-IDP Benjamini–Hochberg-adjusted results and sensitivity estimates from MR-Egger, weighted-median, weighted-mode, and simple-mode analyses are provided in Supplementary Tables S4–S11. CI, confidence interval; WM, white matter. The within-IDP BH correction does not constitute atlas-wide multiplicity control.

**Figure 3:**
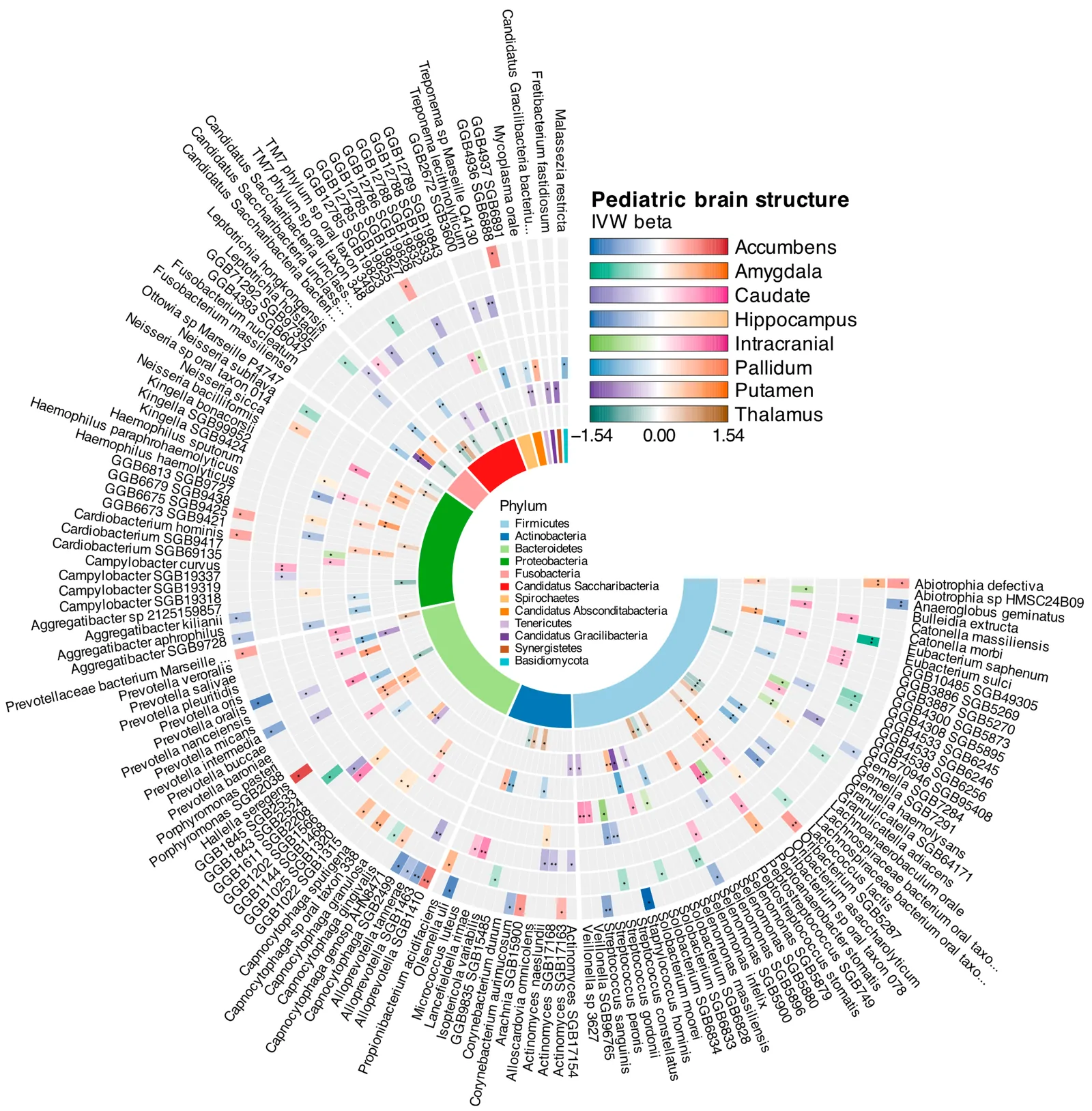
**Genetically proxied oral taxa show nominal associations with childhood brain structure.** Circular heat map of the 146 taxa with at least one nominal inverse-variance weighted (IVW) MR association among 434 taxa tested across eight childhood outcomes: bilateral accumbens, amygdala, caudate, hippocampal, intracranial, pallidal, putaminal, and thalamic volumes. Concentric tracks correspond to the eight outcomes as labeled, and microbial taxa are arranged by phylum around the circumference. Cell color represents the IVW coefficient. *, ** denote unadjusted two-sided IVW *p* < 0.05, *p* < 0.01, respectively. Full IVW and sensitivity results are provided in Supplementary Table S12.

**Figure 4:**
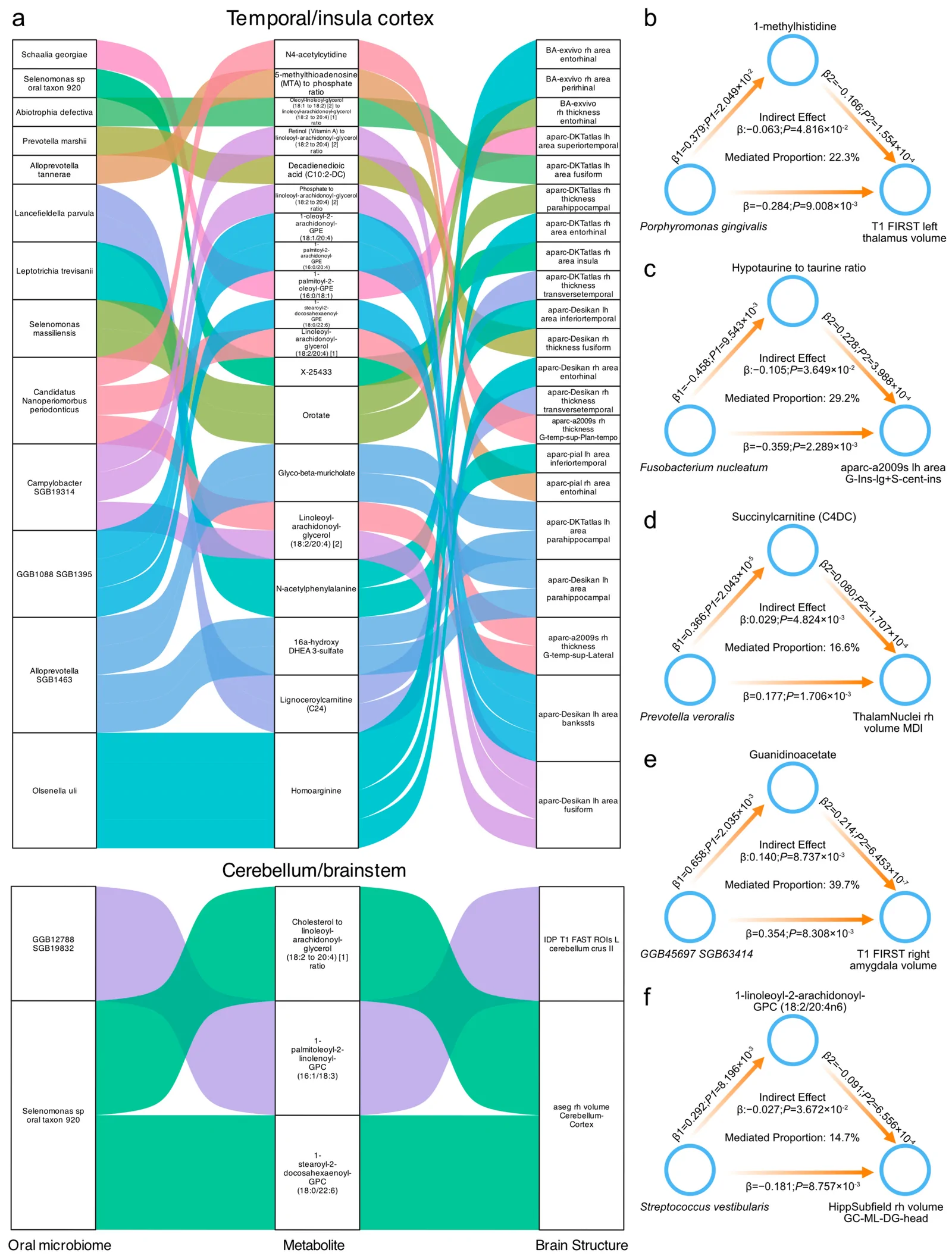
**Plasma metabolites nominate candidate metabolite-linked paths of oral microbiome–brain structural associations.** (**a**) Alluvial diagrams connecting oral microbial exposures, plasma metabolite mediators, and adult brain imaging-derived phenotype (IDP) outcomes in the temporal/insula and cerebellum/brainstem classes. Ribbon colors distinguish individual exposure–mediator–outcome paths. (**b**–**f**) Representative two-step MR indirect-effect models: *Porphyromonas gingivalis*–1-methylhistidine–left thalamic volume (**b**), *Fusobacterium nucleatum*–hypotaurine-to-taurine ratio–left insular surface area (**c**), *Prevotella veroralis*–succinylcarnitine (C4DC)–right mediodorsal-lateral thalamic volume (**d**), GGB45697 SGB63414–guanidinoacetate–right amygdala volume (**e**), and *Streptococcus vestibularis*–1-linoleoyl-2-arachidonoyl-glycerophosphocholine–right granule-cell/molecular-layer dentate-gyrus head volume (**f**). *β*_1_ and *P*_1_ denote the taxon-to-metabolite estimate; *β*_2_ and *P*_2_ denote the metabolite-to-IDP estimate; the horizontal arrow shows the total taxon-to-IDP estimate. The indirect effect was calculated as *β*_1_ × *β*_2_ by the first-order delta method; mediated proportion was the indirect effect divided by the total effect. All paths were selected using nominal criteria and remain exploratory. Thus, 95% CIs for all reported indirect effects and mediated proportions are provided in Supplementary Table S16.

**Figure 5:**
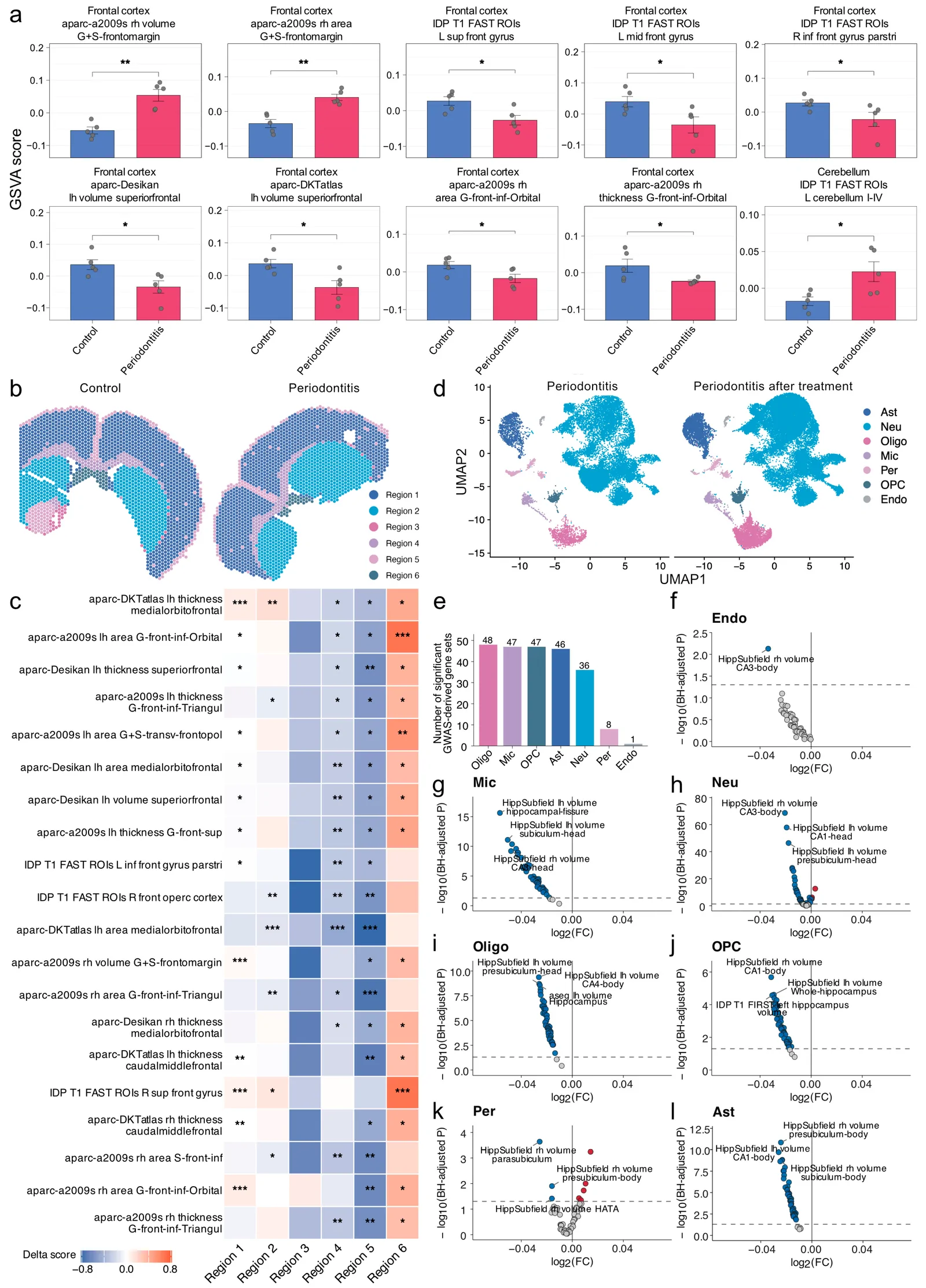
**Periodontitis-associated brain-structure transcriptional programs show regional and cell-level variation.** (**a**) Bulk RNA-sequencing GSVA scores for nine frontal-cortex IDP gene sets and one cerebellar IDP gene set with nominal differences between control and ligature-induced periodontitis mice. Bars show mean ± s.e.m.; points are biological samples (*n* = 5 per group for each tissue). *p* values are from two-sided Wilcoxon rank-sum tests; * *p* < 0.05 and ** *p* < 0.01. (**b**) Spatial transcriptomic maps of one control prefrontal-cortex section (C10PC) and one periodontitis section (L12PC), partitioned into six matched anatomical regions. (**c**) Periodontitis-minus-control differences in standardized scores for 20 frontal IDP gene sets selected for the broadest spatial effects. Two-sided Wilcoxon rank-sum *p* values were BH-adjusted across 924 tests; *, **, and *** denote BH-adjusted *p* < 0.05, BH-adjusted *p* < 0.01, and BH-adjusted *p* < 0.001. (**d**) UMAP representations of 46,547 hippocampal nuclei from eight libraries, shown separately for periodontitis (PD; 17,499 nuclei) and periodontitis after treatment (PDT; 29,048 nuclei), with seven annotated cell types. (**e**) Number of IDP gene sets with global BH-adjusted *p* < 0.05 in each cell type. (**f**–**l**) Cell-type-resolved volcano plots from two-sided nucleus-level Wilcoxon rank-sum tests, with *p* values BH-adjusted across 350 comparisons; the horizontal axis shows log_2_(mean ssGSEA score in PDT/mean ssGSEA score in PD). BH, Benjamini–Hochberg; IDP, imaging-derived phenotype; s.e.m., standard error of the mean; Ast, astrocytes; Endo, endothelial cells; GSVA, gene set variation analysis; Mic, microglia; Neu, neurons; Oligo, oligodendrocytes; OPC, oligodendrocyte precursor cells; Per, pericytes; ssGSEA, single-sample gene set enrichment analysis; UMAP, uniform manifold approximation and projection. Panels (**b**,**c**) have one section per condition and provide descriptive spatial localization. Panels (**e**,**l**) retain exploratory nucleus-level comparisons; their *p* values do not account for within-library dependence and must not be interpreted as population-level significance. Primary library-level testing is reported in Results and Supplementary Table S21.

**Figure 6:**
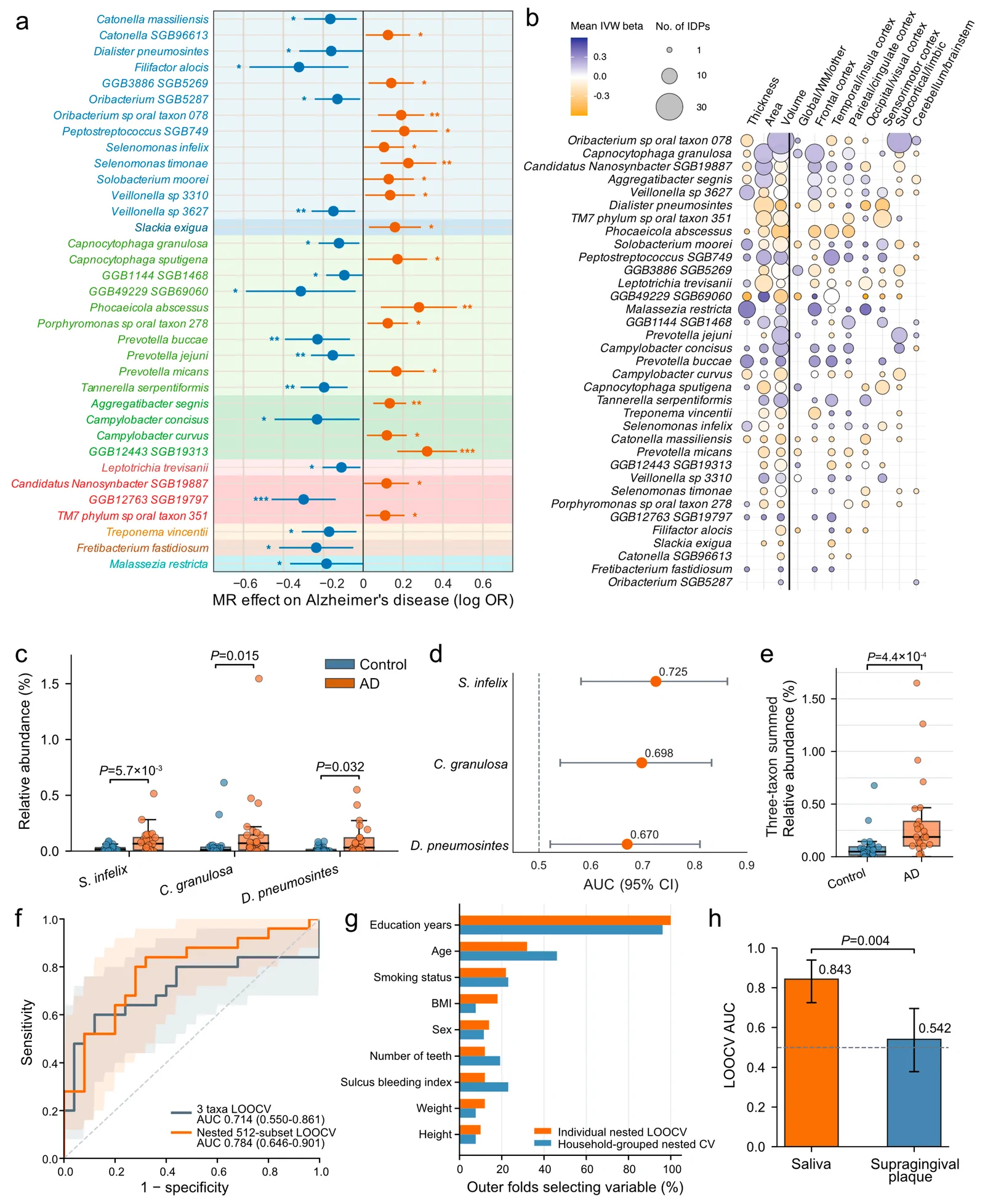
**Brain- and AD-linked oral taxa yield an exploratory proof-of-concept salivary classifier.** (**a**) Forest plot of 35 taxa with nominal inverse-variance weighted (IVW) MR associations with AD and MR-Egger intercept *p* ≥ 0.05. Points show log odds ratios, and bars show 95% confidence intervals (CIs); colors denote phylum. *, **, and *** indicate unadjusted two-sided IVW *p* < 0.05, *p* < 0.01, and *p* < 0.001. (**b**) Adult IDP profiles at raw IVW *p* < 0.01 after the MR-Egger intercept filter (468 descriptive pairs; 384 IDPs). Bubble size denotes counts; color denotes the mean IVW coefficient. (**c**) Salivary relative abundances of *Selenomonas infelix*, *Capnocytophaga granulosa*, and *Dialister pneumosintes* in 25 controls and 25 AD participants. Boxes show medians and interquartile ranges; whiskers extend to 1.5 × the interquartile range; points represent samples. *p* values are from unpaired two-sided Mann–Whitney *U* tests. (**d**) In-cohort fixed-score AUCs for individual taxa, with class-stratified bootstrap 95% CIs. (**e**) Unweighted summed abundance, displayed as in (**c**). (**f**) ROC curves for the fixed three-taxon logistic model (LOOCV AUC, 0.714; 95% CI, 0.550–0.861) and the primary nested clinical-subset selection procedure (AUC, 0.784; 95% CI, 0.646–0.901). Clinical-subset selection occurs entirely within each outer training fold; the three taxa were selected earlier in this cohort. (**g**) Clinical-variable selection frequencies across 50 individual outer folds and 26 household-grouped outer folds. (**h**) Secondary, post-selection same-site refits of the fixed three-taxon-plus-education model in saliva (*n* = 50; AUC, 0.843) and plaque (*n* = 51; AUC, 0.542). AUC, area under the ROC curve; LOOCV, leave-one-out cross-validation; ROC, receiver operating characteristic.

## Data Availability

All analyses used publicly available data. Oral-microbiome GWAS summary statistics are available from the NHGRI–EBI GWAS Catalog under GCST90710236–GCST90710674; adult brain imaging GWAS data under GCST90002426–GCST90003750; childhood brain-volume GWAS data under GCST90296636, GCST90296639, GCST90296642, GCST90296645, GCST90296648, GCST90296651, GCST90296654, and GCST90296657; metabolomic GWAS data under GCST90199621–GCST90201020; and AD GWAS data under GCST90027158. Bulk and spatial transcriptomic data are available under GSE299912/PRJNA1277304 and GSE299911/PRJNA1277302, respectively; single-nucleus data under CRA007555; and full-length oral 16S ribosomal RNA data under PRJNA1243403. All software packages and versions are specified in Methods. Custom R and Python code supporting the analyses is available from the corresponding author upon reasonable request.

## Supplementary Materials

The following supporting information can be downloaded at: https://www.mdpi.com/article/10.3390/microorganisms14102157/s1, The accompanying files contain Table S1: Data sources and study characteristics; Table S2: Anatomical classification of 1325 adult brain imaging-derived phenotypes; Table S3: Genetic instruments for 439 oral microbial traits at *p* < 5 × 10^−6^; Table S4: Oral microbiome MR results for global, white matter, and other adult brain phenotypes; Table S5: Oral microbiome MR results for adult frontal-cortex phenotypes; Table S6: Oral microbiome MR results for adult temporal and insular cortex phenotypes; Table S7: Oral microbiome MR results for adult parietal and cingulate cortex phenotypes; Table S8: Oral microbiome MR results for adult occipital and visual cortex phenotypes; Table S9: Oral microbiome MR results for adult sensorimotor-cortex phenotypes; Table S10: Oral microbiome MR results for adult subcortical and limbic phenotypes; Table S11: Oral microbiome MR results for adult cerebellar and brainstem phenotypes; Table S12: Oral microbiome MR results for eight childhood brain-volume traits; Table S13: Genome-wide significant oral microbial instruments and harmonization eligibility; Table S14: Adult brain MR sensitivity results using oral microbial instruments at *p* < 5 × 10^−8^; Table S15: Childhood brain MR sensitivity results using oral microbial instruments at *p* < 5 × 10^−8^; Table S16: Exploratory metabolite-linked indirect effects and approximate 95% confidence intervals; Table S17: Frontal-cortex IDP GWAS gene-set information; Table S18: Frontal-cortex bulk RNA-sequencing gene set scores and comparisons; Table S19: Cerebellar IDP gene set information and bulk RNA-sequencing comparisons; Table S20: Exploratory spatial region–gene set comparisons; Table S21: Library-level pseudobulk comparisons of hippocampal IDP gene set scores; Table S22: Exploratory nucleus-level hippocampal IDP gene set comparisons; Table S23: Oral microbiome MR results for Alzheimer’s disease; Table S24: Nested validation of 512 clinical-covariate subsets added to the three-taxon classifier; Figure S1: Metric-stratified adult oral microbiome–brain MR associations; Figure S2: Exploratory metabolite-linked paths across six additional anatomical classes; Figure S3: Secondary logistic-regression and XGBoost comparisons for fixed salivary feature sets.
