# Supplementary material for: Integrated Genetic and Multi-Omic Analyses Reveal Oral Microbial Associations with Brain Structure and Alzheimer’s Disease
Source: Microorganisms. 2026 Sep 24;14(10):2157. doi: 10.3390/microorganisms14102157 (PMC13707487; doi:10.3390/microorganisms14102157)
Supplement: Supplementary file 1 [file microorganisms-14-02157-s001.zip › Supplementary figures.pdf]

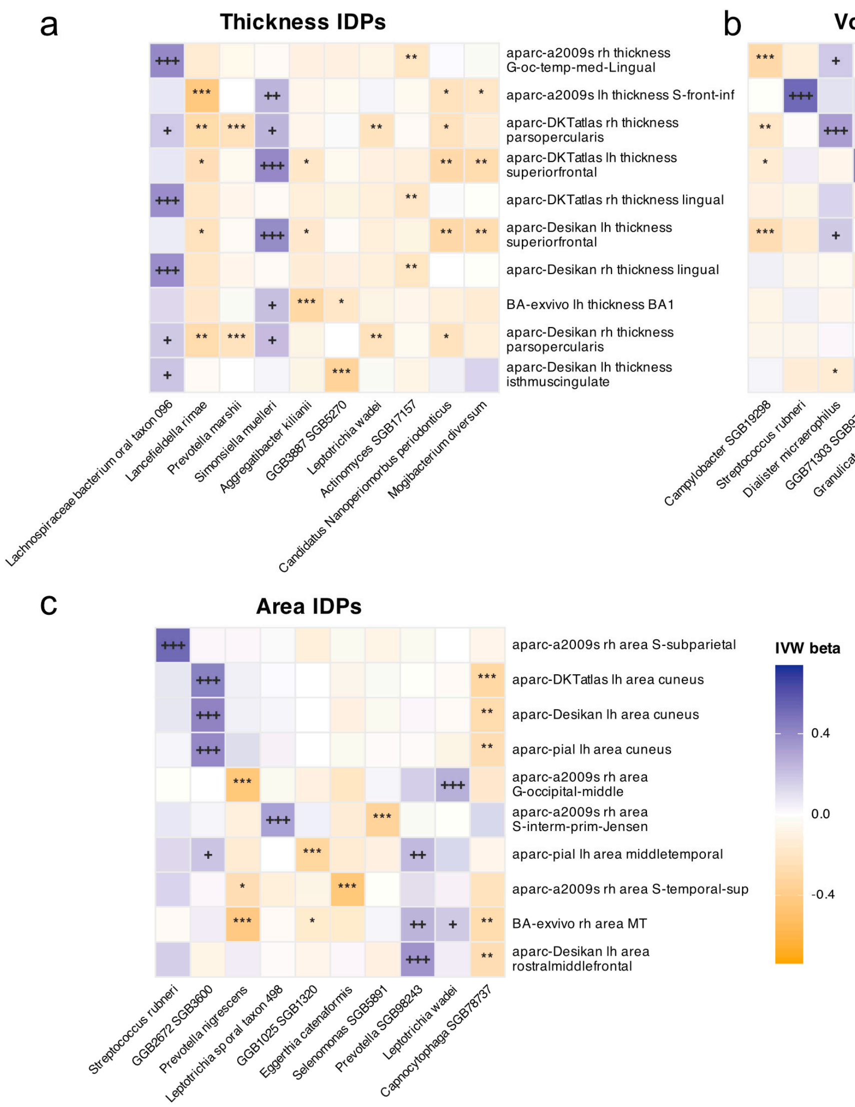

Supplementary Figure S1. Metric-stratified oral microbiome–adult brain structural associations. Heat maps show selected inverse-variance-weighted (IVW) Mendelian randomization (MR) associations for cortical thickness (a), regional volume (b) and surface area (c). Rows are adult brain imaging-derived phenotypes (IDPs) and columns are oral microbial traits. Cell color represents the IVW coefficient (blue, positive; orange, negative). For positive coefficients, +, ++ and +++ denote unadjusted two-sided $P < 0.05$, $P < 0.01$ and $P < 0.001$; for negative coefficients, *, ** and *** denote the same thresholds. Full estimates, within-IDP Benjamini–Hochberg-adjusted P values, heterogeneity tests and MR-Egger intercept tests are provided in Supplementary Tables S4–S11.

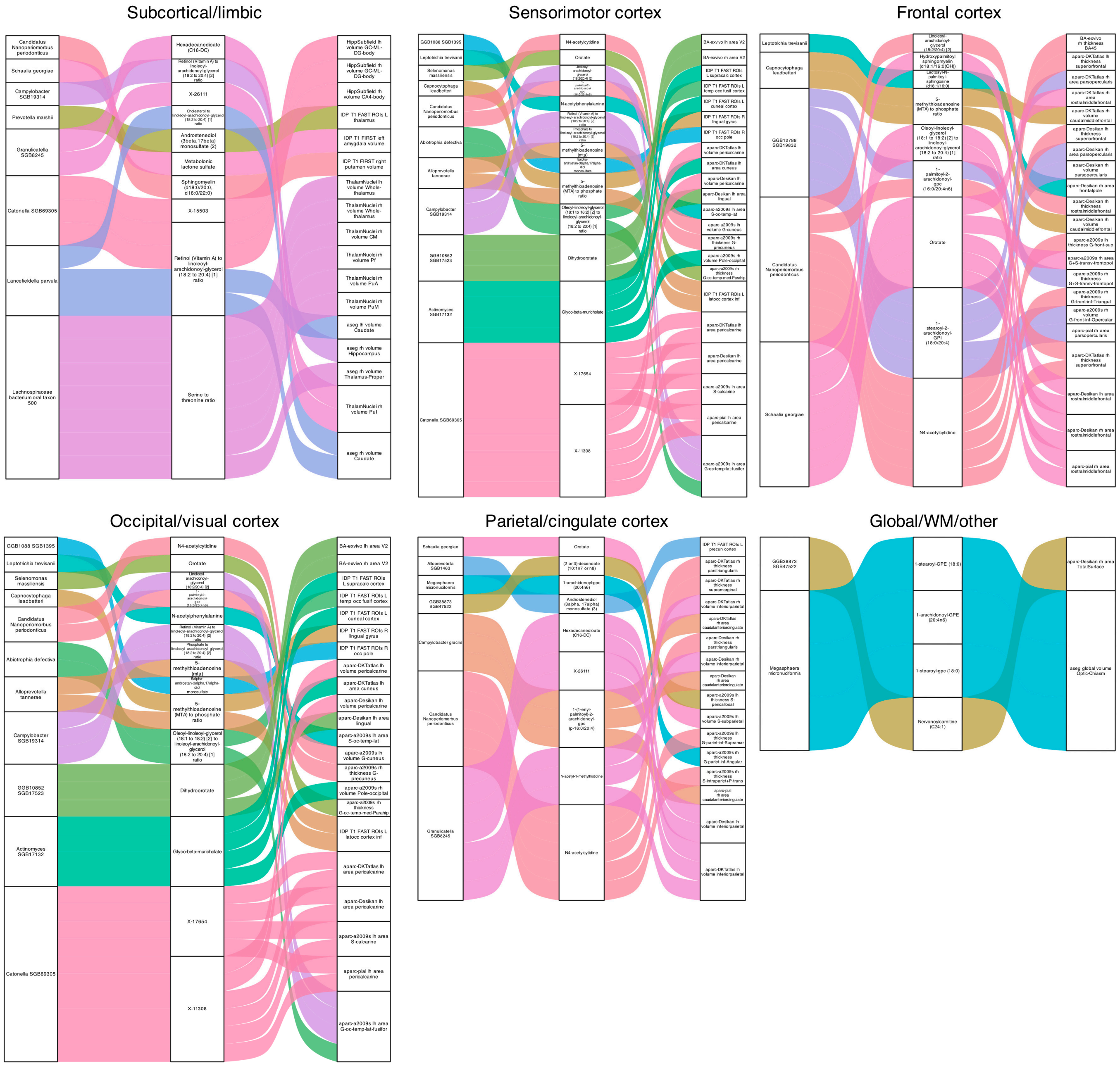

Supplementary Figure S2. Additional candidate metabolite-linked oral microbiome–brain structural pathways. Alluvial diagrams connect oral microbial exposures, plasma metabolite mediators and adult imaging-derived phenotype (IDP) outcomes for the six anatomical classes not shown in Fig. 4: subcortical/limbic, sensorimotor cortex, frontal cortex, occipital/visual cortex, parietal/cingulate cortex and global/white-matter/other traits. Ribbon colors distinguish pathways. All paths are exploratory candidates selected by nominal criteria.

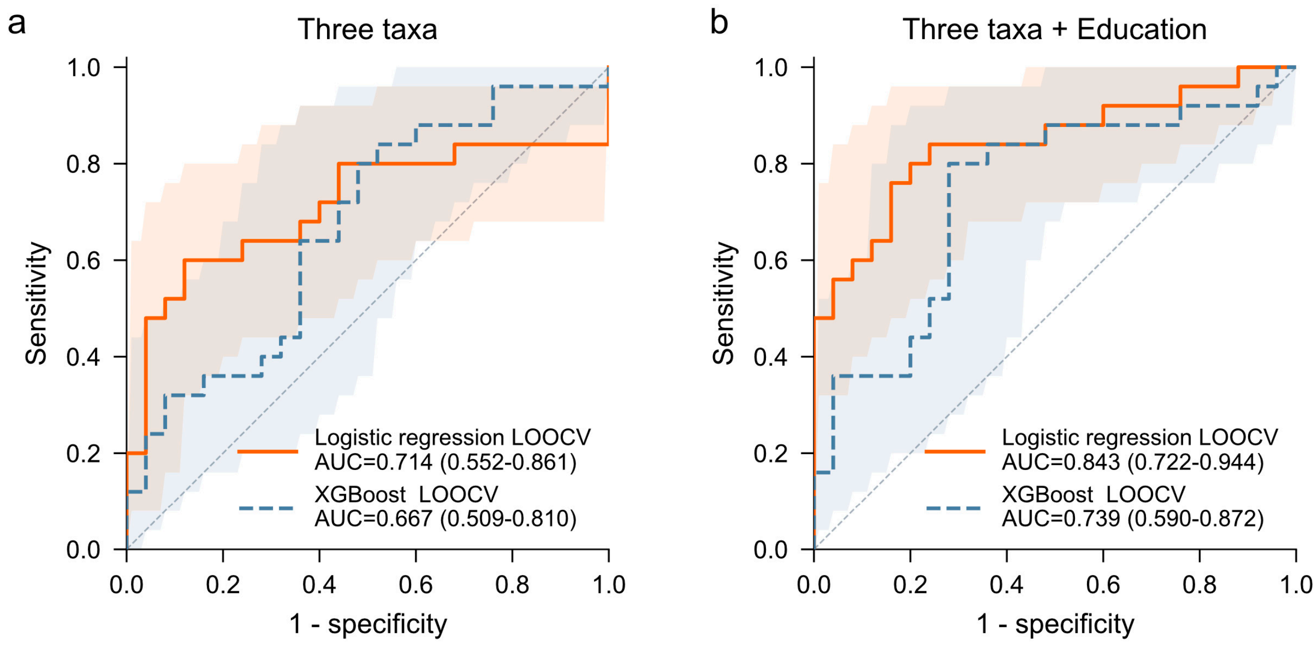

Supplementary Figure S3. Secondary exploratory comparison of logistic regression and XGBoost for fixed salivary feature sets. ROC curves compare logistic regression with XGBoost in 50 saliva samples. a, Three-taxon models using *Selenomonas infelix*, *Capnocytophaga granulosa* and *Dialister pneumosintes*: logistic-regression LOOCV AUC, 0.714 (95% CI, 0.552–0.861); nested-tuned XGBoost AUC, 0.667 (95% CI, 0.509–0.810). b, Three taxa plus education: logistic-regression LOOCV AUC, 0.843 (95% CI, 0.722–0.944); nested-tuned XGBoost AUC, 0.739 (95% CI, 0.590–0.872). AUC, area under the ROC curve; CI, confidence interval; LOOCV, leave-one-out cross-validation.
